# In Utero Antihypertensive Medication Exposure and Neonatal Outcomes: A Data Linkage Cohort Study

**DOI:** 10.1161/HYPERTENSIONAHA.119.13802

**Published:** 2019-12-30

**Authors:** Catherine A. Fitton, Michael Fleming, Markus F.C. Steiner, Lorna Aucott, Jill P. Pell, Daniel F. Mackay, James S. Mclay

**Affiliations:** 1 From the Department of Child Health, Division of Applied Health Sciences, University of Aberdeen, Royal Aberdeen Children’s Hospital, Scotland (C.A.F., M.F.C.S., L.A., J.S.M.); 2 The Institute of Health and Wellbeing, University of Glasgow, Scotland (M.F., J.P.P., D.F.M.).

**Keywords:** antihypertensive agents, child, gestational age, hypertension, pregnancy

## Abstract

Hypertensive disorders during pregnancy are an important risk to mother and fetus, frequently necessitating antihypertensive treatment. Data describing the safety of in utero exposure to antihypertensive treatment is conflicting, with many studies suffering from significant methodological issues, such as inappropriate study design, small sample sizes, and no untreated control group. We conducted a retrospective cohort study using linked routinely collected healthcare records for 268 711 children born 2010–2014 in Scotland to assess outcomes following in utero exposure to antihypertensive medication. We identified a cohort of 265 488 eligible children born over the study period; of which, 2350 were exposed to in utero antihypertensive medication, 4391 exposed to treated late-onset hypertension, and 7971 exposed to untreated hypertension during pregnancy. Untreated hypertension was associated with increased risk of preterm birth (adjusted risk ratio [aRR], 1.15 [99% CI, 1.01–1.30]), low birth weight (aRR, 2.01 [99% CI, 1.72–2.36]) and being small for gestational age (aRR, 1.50 [99% CI, 1.35–1.66]), while in utero antihypertensive exposure was also associated with preterm birth (aRR, 3.12 [99% CI, 2.68–3.64]), low birth weight (aRR, 2.23 [99% CI, 1.79–2.78]), and being small for gestational age (aRR, 2.13 [99% CI, 1.81–2.52]). Late-onset hypertension was also associated with preterm birth (aRR, 2.21 [99% CI, 1.86–2.62]), low birth weight (aRR, 2.06 [99% CI, 1.74–2.43]), and being small for gestational age (aRR, 1.90 [99% CI, 1.68–2.16]). Our results suggest that hypertension is a key risk factor for low birth weight and preterm birth. Although preterm birth may be associated with antihypertensive medication exposure during pregnancy, these associations may reflect increasing hypertension severity necessitating treatment.

## Abstract 2

Supplemental Digital Content is available in the text.

Despite the widespread use of antihypertensive medication during pregnancy, the effects of medication on the fetus are unclear. Published literature is conflicting, with some reporting an increased incidence of preterm birth^1–6^ and low birth weight,^1,3–5^ while others report no increased risk.^7–9^ We recently reviewed antihypertensive use during pregnancy and child outcomes^10^ and identified a lack of good quality studies, with many limited by self-reported medication use,^1,6,11,12^ small study size,^13–15^ and a lack of an untreated comparison group.^1,6,11–13,16–18^ An updated Cochrane review^19^ also assessed trials investigating antihypertensive medication in mild to moderate hypertension during pregnancy and concluded that there was no association between preterm birth or being small for gestational age following medication exposure. Therefore, at present it is not clear whether there is a true association between adverse outcomes and antihypertensive use during pregnancy.

In the United Kingdom, strict guidelines set by The National Institute of Care and Excellence^20^ are followed regarding the treatment of hypertension in pregnant women. National Institute of Care and Excellence defines hypertension as a blood pressure of 140/90 mm Hg or higher, with a blood pressure of 160/110 mm Hg or higher classed as severe hypertension. Antihypertensive agents should be given when a measured blood pressure of 140/90 is exceeded.

Hypertension during pregnancy can fall into several categories: chronic hypertension, indicated by a diagnosis of hypertension or antihypertensive use before 20 weeks, or if unresolved following birth; gestational hypertension, indicated by new onset diagnosis of hypertension after 20 weeks gestation, without proteinuria; preeclampsia, indicated by hypertension occurring after 20 weeks gestation, with or without proteinuria; and preeclampsia superimposed on chronic hypertension, indicated by preeclampsia in the presence of previously diagnosed hypertension.^20^

The aim of this study was to assess the immediate birth outcomes for mother and child after in utero exposure to antihypertensive medication.

## Materials and Methods

Because of the sensitive nature of the data collected for this study, requests to access the dataset from qualified researchers trained in human subject confidentiality protocols may be sent to the Information Services Division at https://www.isdscotland.org/.

This study was approved by the National Health Service Scotland Public Benefit and Privacy Panel for Health and Social Care on the 18th April 2016: reference 1516-0363. Full ethical approval was given by the North West-Greater Manchester South Research Ethics Committee on the 20 April 2016: reference 16/NW/0313.

### Databases

Using the Community Health Index, a unique identifier given to all patients who use the National Health Service in Scotland, individual-level data from 4 Scottish healthcare databases, held by the Information Statistics Division, were linked. The linked databases used were the Scottish Morbidity Record 02 database, which collects data on maternal, obstetric, and child outcomes; the Prescribing Information System which collects information on encashed prescriptions issued by primary care and dispensed from community pharmacies for all Scottish residents.

### Inclusion Criteria, Definitions, and Outcomes

Dispensed antihypertensive medication, excluding propranolol, were used as a surrogate for treated hypertension. Antihypertensive medication included any of the following British National Formulary codes: 2.2 diuretics, 2.4 beta-adrenoceptor blocking drugs, 2.5.2 centrally acting antihypertensive drugs, 2.5.5 drugs affecting the renin-angiotensin system, and 2.6.2 calcium channel blockers. To identify different hypertensive presentations during pregnancy, 4 study groups were defined.

1. Exposure during pregnancy: All women who had a singleton live birth in Scotland between January 2010 and December 2014 and who were dispensed at least one prescription for an antihypertensive medication during the 300 days before birth.
2. Late-onset hypertension: All women who had a singleton live birth in Scotland between January 2010 and December 2014 who were dispensed at least one prescription for an antihypertensive medication during the 60 days after birth, without any prior antihypertensive medication use. This group serves to identify those women who have had late-onset hypertension, or preeclampsia, that have not been treated throughout pregnancy. In the United Kingdom, women with preeclampsia will be started on antihypertensive therapy in secondary care, which is then continued in primary care after discharge. At present, unlike primary care prescribing, there is no national database of inpatient hospital prescriptions. A prescription for antihypertensive medication in the 60 days following birth was, therefore, used as a surrogate marker for preeclampsia or late-onset hypertension that has not resolved after delivery.
3. Untreated hypertensive group: All women who had a singleton birth during the same study period, who had an *International Classification of Diseases-Tenth Revision* (ICD-10) code for one of the following: O10 chronic hypertension, O13 gestational hypertension, O14 preeclampsia, O15 eclampsia, O16 unspecified hypertension, and who were not dispensed antihypertensive medication at any stage during or 60 days after pregnancy (untreated hypertension).
4. Unexposed comparison group: All women who had a singleton birth during the same study period who were not dispensed antihypertensive medication during or 60 days following pregnancy, and who did not have an ICD-10 code for one of the following: O10 chronic hypertension, O13 gestational hypertension, and O16 unspecified hypertension.

Mother-child pairs with a duplicate or missing unique identifier, multiple births, pregnancies ending with abortion, miscarriage or stillbirth, and mothers recorded as younger than 14 years of age were excluded from the study. In the United Kingdom, propranolol is not routinely used in the treatment of hypertension during pregnancy and is not recommended by National Institute of Care and Excellence. The most common cause for prescription of propranolol is anxiety and migraine, which was reflected by the recorded dose range of 10 to 40 mg daily within our cohort. We, therefore, decided that women treated with propranolol should be excluded. Women who were prescribed an ACEi (angiotensin-converting enzyme inhibitor) or an ARB (angiotensin II receptor blocker) during pregnancy were also excluded.

Four immediate birth outcomes for mother and child were studied: preterm birth, low birth weight (adjusted for gestational age), emergency caesarean section, and being small for gestational age. Preterm birth was defined as birth on or before 36 weeks gestation. Low birth weight was defined as a birth weight of <2500 g. Being small for gestational age was defined as having a birth weight below the 10th percentile of the population. Population percentiles were calculated from the complete cohort as this represents the full Scottish population born 2010 to 2014.

The outcome measures were reported either as dichotomous variables (preterm birth, low birth weight, emergency caesarean section), or as continuous variables (gestational age, birth weight). The Scottish Morbidity Record 02 data set provided data on the mother’s ethnicity, smoking status, alcohol and illicit drug use during pregnancy, previous stillbirth parity, maternal body mass index, maternal age, diabetes mellitus status, and Scottish Index of Multiple Deprivation quintile score, which was used as a marker of area socioeconomic deprivation. These variables were previously identified using a directed acyclic graphs process^21^ and so were treated as potential confounders.

### Statistical Analyses

The characteristics of mothers exposed to antihypertensive medication during pregnancy were described using appropriate summary statistics depending on the data type and distribution.

Where data were missing, multiple imputation chained equations were performed, using the MI impute function in STATA, to create 30 data sets.^22^ The following variables were imputed: body mass index, Scottish Index of Multiple Deprivation, smoking, drug use, alcohol use, previous stillbirths, and parity. Body mass index was imputed by predictive mean matching with a K-nearest neighbors of 10, while the other variables were imputed by either multinomial, ordinal, or logistic regression. Rubin’s rules were used to combine the results of the imputed data sets to provide one overall estimate, with a CI adjusted for uncertainty.

Poisson regressions with log links were performed for all dichotomous outcomes and linear regressions for continuous variables. Regressions were adjusted for relevant confounders as identified by directed acyclic graphs and then a stepwise model selection using the F test with a *P* value cutoff of 0.2. Models were adjusted for body mass index, Scottish Index of Multiple Deprivation, smoking, diabetes mellitus, previous stillbirths, parity, illicit drug use, recorded diagnosis of preeclampsia during the current pregnancy, maternal age, and interaction terms identified. All outcomes were modeled individually and are provided in Tables 1 through 5 and Tables S2 through S5 in the online-only Data Supplement. Univariate and multivariate regression are presented with risk ratios (RR), adjusted RRs (aRR), and 99% CI. Analysis of data was performed in STATA MP, version 14.1 (StataCorp).

**Table 1.**
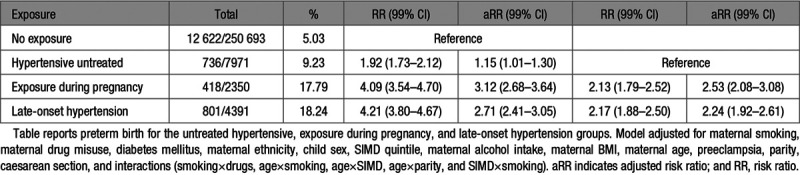
Preterm Birth Following In Utero Antihypertensive Exposure

**Table 2.**
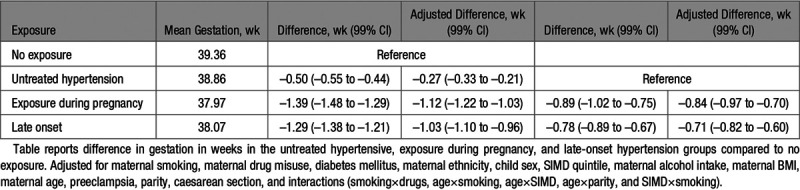
Gestation (Weeks) Linear Regression Results

**Table 3.**
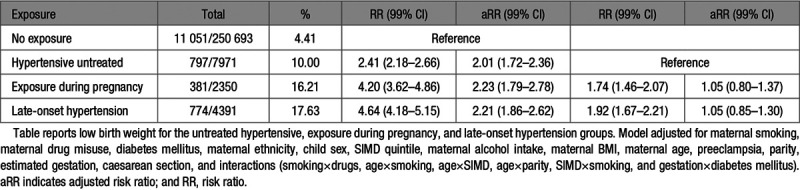
Low Birth Weight Following In Utero Antihypertensive Exposure

**Table 4.**
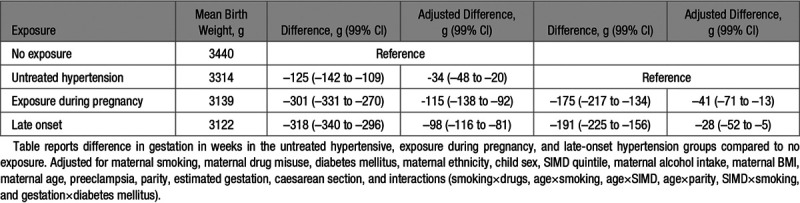
Birthweight (g) Linear Regression Results

**Table 5.**
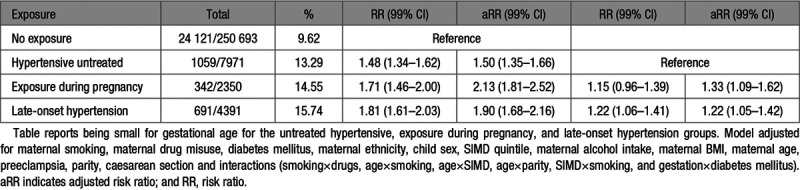
Small for Gestational Age Following In Utero Antihypertensive Exposure

## Results

SMR 02 identified a total of 271 647 live births to 245 438 women during 2010 to 2014. Of these, 268 711 were singleton live births to 226 840 women aged >14 years: 39 949 women had ≥2 singleton deliveries over the period. After exclusion of 3223 mother-child pairs prescribed propranolol and 83 mother-child pairs prescribed an ACEI or ARB during pregnancy, 265 405 women-child pairs were eligible for inclusion in the study. Demographics of the study cohort are reported in the data S1, along with missing data totals.

### Exposure to Antihypertensive Medication

A total of 2350 offspring were exposed to antihypertensive medication during pregnancy, and 4391 offspring were born to women in the late-onset hypertension group. In the unexposed hypertensive group, a total of 7971 offspring were identified and 252 598 offspring in the unexposed comparison group. The majority of offspring were exposed to a β-blocker only (58.66%, 4003 children), calcium channel blockers only (8.18%, 558 children), or a combination of >1 antihypertensive medication (20.53%, 1403 children). The remainder of antihypertensive exposure was a mixture of loop diuretics (5.96%, 407 children), centrally acting antihypertensives (2.87%, 196 children), and thiazides (1.99%, 136 children).

### Maternal Outcomes

A significantly increased risk of emergency caesarean section was associated with untreated hypertension (aRR, 1.63 [99% CI, 1.51–1.75]), exposure during pregnancy (aRR, 1.45 [99% CI, 1.27–1.64]), and late-onset hypertension (aRR, 2.16 [99% CI, 1.98–2.37]), compared with the unexposed comparison group (S2). When compared with the untreated hypertensive group, antihypertensive exposure during pregnancy was associated with a slight decreased risk of emergency caesarean section (aRR, 0.83 [99% CI, 0.71–0.96]), while late-onset hypertension was associated with increased risk (aRR, 1.24 [99% CI, 1.11–1.39]; S2).

### Neonatal Outcomes

#### Preterm Birth

Risk of preterm birth was significantly associated with untreated hypertension (aRR, 1.15 [99% CI, 1.01–1.30]), exposure during pregnancy (aRR, 3.12 [99% CI, 2.68–3.64]), and late-onset hypertension (aRR, 2.71 [99% CI, 2.41–3.05]), compared with the unexposed comparison group (Table 1). When compared with the untreated hypertensive group, both exposure during pregnancy (aRR, 2.53 [99% CI, 2.08–3.08]) and late-onset hypertension (aRR, 2.24 [99% CI, 1.92–2.61]) were associated with increased risk of preterm birth (Table 1). After linear regression analysis of gestational age in weeks, exposure during pregnancy demonstrated the largest difference in gestational age at birth (adjusted difference in weeks, –1.12 [99% CI, –1.22 to –1.03]), followed by late-onset hypertension (adjusted difference in weeks, –1.03 [99% CI, –1.10 to –0.96]) and untreated hypertension (adjusted difference in weeks, –0.27 [99% CI, –0.33 to –0.21; Table 2).

Exposure to any single antihypertensive drug group during pregnancy (β-blocker, calcium channel blocker, centrally acting antihypertensive) or >1 group of antihypertensive medications was significantly associated with increased risk of preterm birth (S3). Treatment started in any trimester was associated with increased risk of preterm birth (S3).

#### Low Birth Weight

Following adjustment for gestational age, significantly increased risk of low birthweight was associated with untreated hypertension (aRR, 2.01 [99% CI, 1.72–2.36]), exposure during pregnancy (aRR, 2.23 [99% CI, 1.79–2.78]), and late-onset hypertension (aRR, 2.21 [99% CI, 1.86–2.62]) compared with the unexposed comparison group (Table 3). There was no significant difference in the risk of low birth weight between the untreated hypertensive group, the exposed during pregnancy, and late-onset hypertension groups (Table 3).

After linear regression analysis of birth weight in grams, exposure during pregnancy demonstrated the largest difference in birth weight (adjusted difference in grams, –115 [99% CI, –138 to –92]), followed by late-onset hypertension (adjusted difference in grams, –98 [99% CI, –116 to –81]) and untreated hypertension (adjusted difference in grams, –34 [99% CI, –48 to –20]; Table 4). Women exposed to an antihypertensive in every trimester of pregnancy or during the third trimester only had a significantly increased risk of having a low birth weight baby (S4).

After assessment of individual drug groups, exposure to a β-blocker (aRR, 2.46 [99% CI, 1.75–3.45]) or >1 class of antihypertensive medication (aRR, 2.45 [99% CI, 1.77–3.38]) at any time during pregnancy was associated with significantly increased risk of low birth weight (S4).

### Small for Gestational Age

There was an increased risk for being born small for gestational age following antihypertensive exposure during pregnancy (aRR, 2.13 [99% CI, 1.81–2.52]), late-onset hypertension (aRR, 1.90 [99% CI, 1.68–2.16]), and untreated hypertension (aRR, 1.50 [99% CI, 1.35–1.66]) when compared with the untreated normotensive group (Table 5). When the antihypertensive exposure during pregnancy and late-onset hypertension groups were compared with the untreated hypertensive group, there was an increased risk of being small for gestational age (aRR, 1.33 [99% CI, 1.09–1.62]; aRR, 1.22 [99% CI, 1.05–1.42]), respectively Table 5.

Antihypertensive exposure in trimester 3 only (aRR, 2.51 [99% CI, 1.93–3.31]) and throughout pregnancy (aRR, 2.72 [99% CI, 2.06–3.58]) was associated with increased risk of being small for gestational age (S5). When assessing individual drug exposure during pregnancy, exposure to a β-blocker only (aRR, 2.22 [99% CI, 1.77–2.80]) or >1 mediation over the pregnancy period (aRR, 2.37 [99% CI, 1.86–3.03]) was associated with increased risk of being small for gestation (S5). Individual β-blockers were all associated with increased risk, with the largest increase following atenolol exposure (aRR, 3.83 [99% CI, 2.03–7.21]; S5).

## Discussion

In this Scottish cohort of 268 711 children born between 2010 and 2014, we demonstrated increased risk of preterm birth, low and extremely low birth weight, being small for gestational age, and emergency caesarean section following exposure to any antihypertensive medication during pregnancy. However, when compared with the untreated hypertensive group, preterm birth and being small for gestational age remained significant. Furthermore, compared with the untreated hypertensive group, there was an increased risk of emergency caesarean section after late-onset hypertension, while we report a decreased risk following antihypertensive exposure during pregnancy. These findings suggests that a mix of hypertension itself, and exposure to antihypertension medication, is responsible for the observed increased risks of this study.

While there is previous literature reporting an increased risk of adverse outcomes, such as preterm birth and low birth weight following in utero antihypertensive exposure,^2,4–6,17,23^ the majority of these studies were of small size, had a poor study design, such as inappropriate control groups, methodologies with residual confounding, and lacked an untreated hypertensive group, leading to further possible confounding. We have reported outcomes following in utero antihypertensive exposure compared to that of an unexposed comparison group and an untreated hypertensive group, allowing us to differentiate between the risks due to underlying hypertension and medication exposure.

We report increased risk of preterm birth in the offspring of those exposed during pregnancy, late-onset hypertension, and in the untreated hypertension groups when compared with the unexposed comparison group. When the untreated hypertensive group was used as a comparison, this association remained for both the exposed during pregnancy and late onset hypertension groups. Our observations suggest that hypertension per se increases the risk of preterm birth and that this risk increases with medication, although the association with medication likely reflects increasing hypertension severity rather than a direct drug effect.

We observed significantly increased risk of low birth weight, for all exposure groups including the untreated hypertensive group, compared with the unexposed comparison group. However, when antihypertensive exposure groups (during pregnancy and late-onset hypertension) were compared with the untreated hypertensive group, there was no increased risk of low birth weight, indicating that the risk of low birth weight is likely due to hypertension rather than medication exposure.

While several studies suffering from methodological limitations^2,3,5,6,23^ have reported an increased risk of preterm birth and low birth weight associated with antihypertensive medication exposure, our results suggest that the increased risk of preterm birth and low birth weight is likely due to hypertension severity rather than medication exposure.

In the Scottish population, the emergency caesarean rate is 15.9% for the general population,^24^ up to 40% in women with chronic hypertension,^25^ and between 30% and 60% in women with preeclampsia.^26,27^ We observed an emergency caesarean rate of 14.73% in our unexposed comparison group, 30.55% in the untreated hypertension group, 28.03% in the exposed group, and 37.90% in the late-onset hypertension group, which are in line with published levels thus strengthening our group selection.

### Strengths of the Study

The use of Scotland-wide data sets, containing routinely collected healthcare data, permitted identification a large, nonselective cohort covering all eligible pregnancies in Scotland over a 5-year period. This approach obviated the possibility of selection or recruitment bias and provided sufficient statistical power to undertake sub-group analyses. Linkage of routinely collected healthcare data for offspring provided information on a wide range of child outcomes from birth to 30-month follow-up. Using dispensing data to determine drug exposure rather than issued prescriptions or self-reported medication use reduced the risk of reporting bias.

### Limitations of Study

Several assumptions were made in this study: use of an antihypertensive medication was solely for treatment of hypertension; women who did not have an ICD-10 code recorded in the Scottish Morbidity Record 02 data and who did not have antihypertensive medication dispensed were normotensive; and exposure following birth was indicative of late-onset hypertension or preeclampsia. As detailed clinical information was not available, we could not identify the severity of hypertension in affected women. While we cannot say whether hypertension was severe, we can use antihypertensive medication use as a proxy in this case, particularly if women are on >1 antihypertensive medication during pregnancy, indicating a greater need for blood pressure control. Due to the number of outcomes assessed, multiple testing was a potential issue. This was dealt with by increasing the threshold of significance and using 99% CIs. However, this poses a further limitation in that we may have missed significant results that do not meet these strict significance levels. Furthermore, due to the data-linkage cohort study design, it is not possible to guarantee coherence to medication regimes. We have attempted to limit this confounding by only using dispensed prescription data generated following the woman obtaining the medication from a pharmacy, rather than the prescription issued by a doctor.

### Perspectives

We demonstrated an increased risk of preterm birth, low birth weight, small for gestational age, and emergency caesarean section in the offspring of both treated and untreated hypertensive women. Rather than treatment with antihypertensive medication, hypertension severity may dictate the risk of preterm birth, while underlying hypertension may be the cause of low birth weight and small for gestational age.

## Acknowledgments

We acknowledge the support from the Farr Institute @ Scotland. The Farr Institute @ Scotland is supported by a 10-funder consortium: Arthritis Research UK, the British Heart Foundation, Cancer Research UK, the Economic and Social Research Council, the Engineering and Physical Sciences Research Council, the Medical Research Council, the National Institute of Health Research, the National Institute for Social Care and Health Research (Welsh Assembly Government), the Chief Scientist Office (Scottish Government Health Directorates), the Wellcome Trust, (MRC grant number MR/K007017/1). J.S. Mclay had the original concept. All authors agreed the study design. C.A. Fitton undertook data analysis and cleaning. All authors interpreted the results. C.A. Fitton and M.F.C. Steiner drafted the article, and all other authors contributed revisions. All authors reviewed and approved the final version of the manuscript. J.S. Mclay is guarantor for the study.

## Sources of Funding

Funding was provided by the FARR Institute at Scotland. The Farr Institute @ Scotland is supported by a 10-funder consortium: Arthritis Research UK, the British Heart Foundation, Cancer Research UK, the Economic and Social Research Council, the Engineering and Physical Sciences Research Council, the Medical Research Council, the National Institute of Health Research, the National Institute for Social Care and Health Research (Welsh Assembly Government), the Chief Scientist Office (Scottish Government Health Directorates), the Wellcome Trust, (MRC Grant No: MR/K007017/1).

## Disclosures

None.

## Supplementary Material



## Novelty and Significance

### What Is New?

- This is the first Scotland-wide study of blood pressure medications taken in pregnancy and the effects on the baby.

### What Is Relevant?

- Women who had high blood pressure requiring treatment during pregnancy were more likely to deliver earlier. All women with hypertension, regardless of whether treated or untreated, had a similar risk of having low birth weight babies.
- Emergency caesarean section was decreased in those treated with medication during pregnancy compared to those untreated.

### Summary

This is the first Scotland-wide study looking at blood pressure medication taken during pregnancy. High blood pressure, whether treated or untreated, was associated with a range of poorer outcomes in the child.
