# Supplementary figures and images for: In Utero Antihypertensive Medication Exposure and Neonatal Outcomes: A Data Linkage Cohort Study
Source: Hypertension. 2019 Dec 30;75(3):628–33. doi: 10.1161/HYPERTENSIONAHA.119.13802 (PMC8032216; doi:10.1161/HYPERTENSIONAHA.119.13802)

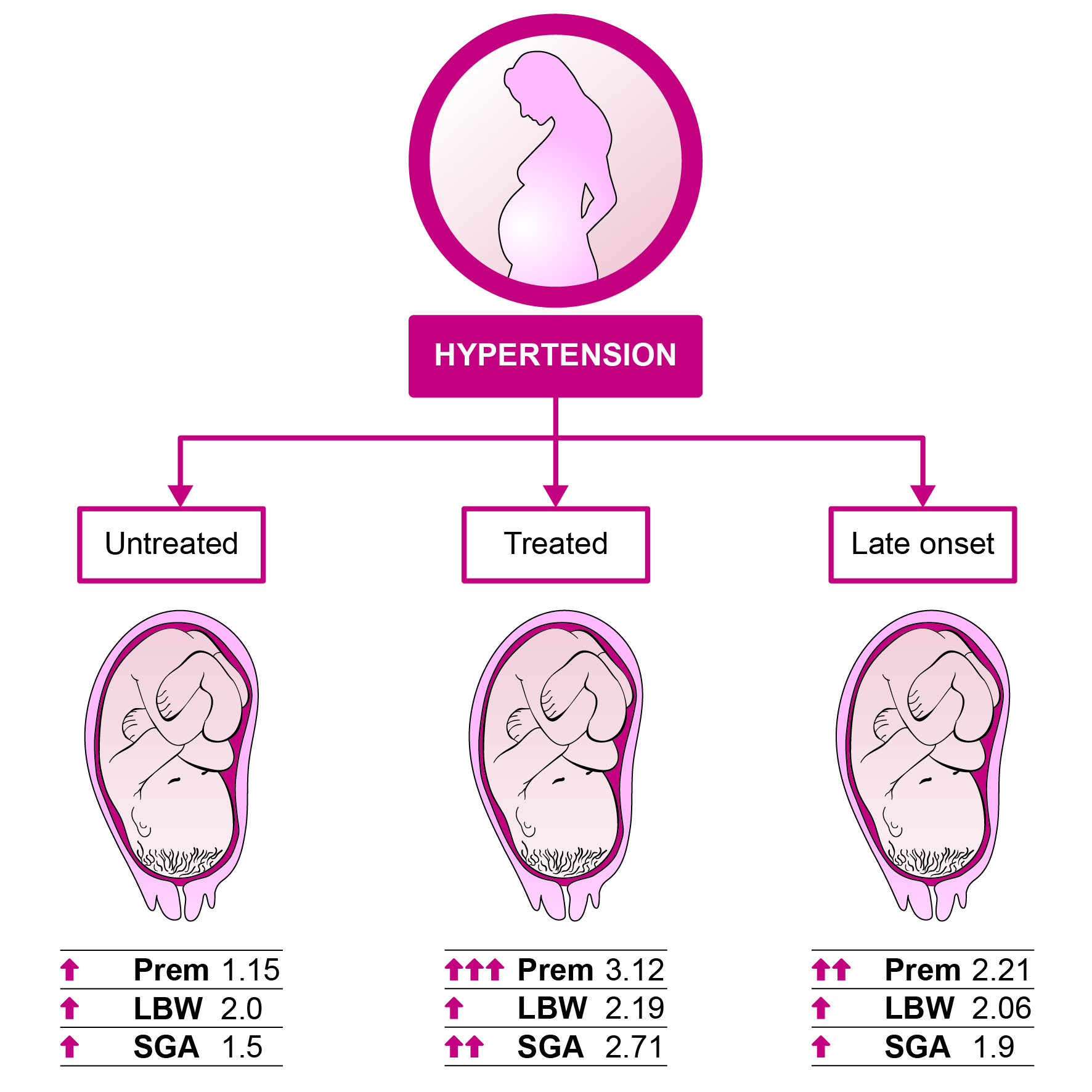

Supplement: Supplementary file 1 [file hyp-75-628-s001.jpg]
