# Supplementary material for: In Utero Antihypertensive Medication Exposure and Neonatal Outcomes: A Data Linkage Cohort Study
Source: Hypertension. 2019 Dec 30;75(3):628–33. doi: 10.1161/HYPERTENSIONAHA.119.13802 (PMC8032216; doi:10.1161/HYPERTENSIONAHA.119.13802)
Supplement: Supplementary file 2 [file hyp-75-628-s002.docx]

**MS TITLE: In utero antihypertensive medication exposure and neonatal outcomes: A data linkage cohort study**

**Supplemental tables 1-5**

Authors: Dr Catherine A FITTON^a^, PhD; Dr Michael FLEMING^b^, PhD; Dr Markus FC STEINER^a^, PhD; Dr Lorna AUCOTT^a^, PhD; Dr Jill P PELL^b^, MD; Dr Daniel F MACKAY^b^, PhD; Dr James S MCLAY^a^, PhD

^a^The Department of Child Health, University of Aberdeen, Royal Aberdeen Children’s Hospital, Westburn Road, Aberdeen AB25 2ZG, Scotland

^b^The Institute of Health and Wellbeing, University of Glasgow, 1 Lilybank Gardens, Glasgow G12 8RZ, Scotland

**Conflicts of Interest and Source of Funding:** No conflicts of interest to declare. Medical Research Council and Farr (Scotland) Institute.

**Corresponding author**: Dr James McLay (j.mclay@abdn.ac.uk), The Department of Child Health, University of Aberdeen, Royal Aberdeen Children’s Hospital, Westburn Road, Aberdeen AB25 2ZG, Scotland, Phone: +44 (0) 1224 438452

**S1. Cohort demographics with antihypertensive exposure groups. Total % may not equal 100% due to rounding**

| Characteristics | |  | | | |  | |  | |  | |
| --- | --- | --- | --- | --- | --- | --- | --- | --- | --- | --- | --- |
|  | Unexposed comparison | | | | Untreated hypertension | | | Exposure during pregnancy | | Late onset hypertension | |
| Total women/child pairs: | 250,693 | | | | 7,971 | | | 2,350 | | 4,391 | |
| Characteristic | N | | % | | N | | % | N | % | N | % |
| **Age (years)** |  | |  | |  | |  |  | |  | |
| Median | 29 | | | | 29 | | | 33 | | 31 | |
| Mean | 29.1 | | | | 28.9 | | | 32.5 | | 31.1 | |
| IQR | 25-33 | | | | 24-33 | | | 29-37 | | 27-35 | |
| Missing | 0 | | | | 0 | | | 0 | | 0 | |
| **BMI** |  | |  | |  | |  |  | |  | |
| Median | 24.6 | | | | 26.4 | | | 28.4 | | 27 | |
| Mean | 25.7 | | | | 27.3 | | | 28.9 | | 27.8 | |
| IQR | 22-28.5 | | | | 23.3-30.8 | | | 24.4-33.0 | | 23.8-31.2 | |
| Missing | 33,036 | | | 13.2 | 951 | | 11.9 | 442 | 18.8 | 571 | 13.0 |
| **Previous stillbirths** |  | |  | |  | |  |  |  |  |  |
| Yes | 1,305 | | 0.5 | | 26 | | 0.3 | 38 | 1.6 | 22 | 0.5 |
| Missing | 1027 | | | 0.4 | 10 | | 0.1 | 16 | 0.7 | 10 | 0.2 |
| **Parity** |  | |  | |  | |  |  | |  | |
| 0 | 108,913 | | 43.4 | | 4,998 | | 62.7 | 1,037 | 44.1 | 2,555 | 58.2 |
| 1 | 88,416 | | 35.3 | | 1,916 | | 24.0 | 713 | 30.3 | 1,110 | 25.3 |
| 2 | 34,325 | | 13.7 | | 668 | | 8.4 | 345 | 14.7 | 432 | 9.8 |
| 3+ | 16,731 | | 6.7 | | 352 | | 4.4 | 221 | 9.4 | 261 | 5.9 |
| Missing | 2,308 | | | 0.9 | 37 | | 0.5 | 34 | 1.5 | 33 | 0.8 |
| **Antenatal steroids** |  | |  | |  | |  |  | |  | |
| Yes | 6,785 | | 2.7 | | 429 | | 5.4 | 257 | 10.9 | 469 | 10.7 |
| Missing | 1,598 | | | 0.6 | 95 | | 1.2 | 58 | 2.5 | 100 | 2.3 |
| **Caesarean section** |  | |  | |  | |  |  | |  | |
| Planned | 30,498 | | 12.2 | | 452 | | 5.7 | 525 | 22.3 | 543 | 12.4 |
| Emergency | 36,917 | | 14.7 | | 2,435 | | 30.5 | 656 | 27.9 | 1,664 | 37.9 |
| Missing | 0 | |  | | 0 | |  | 0 |  | 0 |  |
| **Diabetes** |  | |  | |  | |  |  | |  | |
| Yes (pre-existing) | 1,279 | | 0.5 | | 108 | | 1.4 | 106 | 4.5 | 76 | 1.7 |
| Yes (gestational) | 3,812 | | 1.5 | | 209 | | 2.6 | 128 | 5.4 | 151 | 3.4 |
| Yes (unknown) | 323 | | 0.1 | | 5 | | 0.1 | 10 | 0.4 | 9 | 0.2 |
| Missing | 6,145 | | | 2.5 | 106 | | 1.3 | 53 | 2.3 | 87 | 2.0 |
| **Drug Misuse** |  | |  | |  | |  |  |  |  |  |
| Yes | 4,313 | | 2.10 | | 107 | | 1.34 | 28 | 1.2 | 57 | 1.52 |
| Missing | 45,050 | | | 18.0 | 1,205 | | 15.1 | 410 | 17.4 | 636 | 14.5 |
| **Alcohol (units/week)** |  | |  | |  | |  |  | |  | |
| 0 – 2 | 199.291 | | 79.5 | | 6,220 | | 78.0 | 1,869 | 79.5 | 3,595 | 81.9 |
| 3 – 6 | 3,770 | | 1.5 | | 200 | | 2.5 | 38 | 1.6 | 71 | 1.6 |
| >6 | 3,857 | | 1.5 | | 165 | | 2.1 | 40 | 1.7 | 60 | 1.4 |
| Missing | 43,775 | | | 17.5 | 1,386 | | 17.4 | 403 | 17.1 | 665 | 15.1 |
| **Smoking** |  | |  | |  | |  |  | |  | |
| Yes | 45,222 | | 18.0 | | 935 | | 11.7 | 283 | 12.0 | 516 | 11.8 |
| Missing | 12,994 | | | 5.2 | 342 | | 4.3 | 146 | 6.2 | 208 | 4.7 |
| **SIMD Quintile** |  | |  | |  | |  |  | |  | |
| 1 | 65,763 | | 26.2 | | 2,021 | | 25.4 | 618 | 26.3 | 1,045 | 23.8 |
| 2 | 53,264 | | 21.2 | | 1,758 | | 22.1 | 524 | 22.3 | 925 | 21.1 |
| 3 | 47,875 | | 19.1 | | 1,524 | | 19.1 | 480 | 20.4 | 901 | 20.5 |
| 4 | 43,981 | | 17.5 | | 1,455 | | 18.3 | 416 | 17.7 | 858 | 19.5 |
| 5 | 39,138 | | 15.6 | | 1,199 | | 15.0 | 308 | 13.1 | 652 | 14.8 |
| Missing | 672 | | | 0.3 | 14 | | 0.2 | 4 | 0.2 | 10 | 0.3 |
| **Ethnic Group** |  | |  | |  | |  |  |  |  |  |
| White | 155,338 | | 62.0 | | 5,225 | | 65.6 | 1,538 | 65.4 | 2,768 | 63.0 |
| Other | 12,370 | | 4.9 | | 272 | | 3.4 | 109 | 4.6 | 206 | 4.7 |
| Missing | 82,985 | | | 33.1 | 2,474 | | 31.0 | 703 | 29.9 | 1,417 | 32.3 |
| **Gender of Birth** |  | | |  |  | |  |  |  |  |  |
| Male | 128,538 | | | 51.3 | 4,229 | | 53.1 | 1,178 | 50.1 | 2,206 | 50.2 |

**S2. Emergency caesarean section risk following in utero antihypertensive exposure. Table reports emergency caesarean section results for the untreated hypertensive, exposure during pregnancy and late onset hypertension groups. Model adjusted for: maternal smoking, maternal drug misuse, diabetes, maternal ethnicity, child sex, SIMD quintile, maternal alcohol intake, maternal BMI, maternal age, preeclampsia, parity, estimated gestation and interactions (smoking*drugs, age*smoking, age*SIMD, age*parity, SIMD*smoking, gestation*diabetes RR = Risk ratio; 99% CI = 99% confidence interval; aRR = adjusted risk ratio.**

| **Exposure** | **Total** | **%** | **RR (99% CI)** | **aRR (99% CI)** | **RR (99% CI)** | **aRR (99% CI)** |
| --- | --- | --- | --- | --- | --- | --- |
| No exposure | 36,917/250,693 | 14.73 | Reference | |  | |
| Hypertensive untreated | 2,435/7,971 | 30.55 | 2.55 (2.39-2.72) | 1.63 (1.51-1.75) | Reference | |
| Exposure during pregnancy | 656/2,350 | 27.91 | 2.24 (1.99-2.53) | 1.45 (1.27-1.64) | 0.88 (0.77-1.01) | 0.83 (0.71-0.96) |
| Late onset hypertension | 1,664/4,391 | 37.90 | 3.53 (3.26-3.83) | 2.16 (1.98-2.37) | 1.39 (1.25-1.53) | 1.24 (1.11-1.39) |

**S3. Sub-analyses of Preterm birth following in utero antihypertensive exposure. Table reports exposure to any antihypertensive started during the first trimester onwards, second trimester onwards and third trimester onwards, and exposure to centrally acting antihypertensive only, beta blocker only, calcium channel blocker only, or more than one class of medication. Adjusted for: Maternal BMI, maternal diabetes, parity, smoking status, maternal age, preeclampsia, SIMD quintile, drug misuse, alcohol intake, previous stillbirths and interactions (diabetes*gestation, age*SIMD, age*smoking, alcohol*drugs). OR = Risk ratio; 99% CI = 99% confidence interval; aRR = adjusted risk ratio.**

| **Preterm Birth** | **Total** | **Percentage** | **RR (99% CI)** | **aRR (99% CI)** |
| --- | --- | --- | --- | --- |
| No exposure | 12,622/250,693 | 5.03 | Reference | |
| **Trimesters** | | | | |
| Trimester 1 only | 64/439 | 14.58 | 3.22 (2.27-4.56) | 2.65 (1.84-3.82) |
| Trimester 2 only | 15/100 | 15.00 | 3.33 (1.62-6.85) | 2.41 (1.12-5.17) |
| Trimester 3 only | 156/768 | 20.31 | 4.81 (3.81-6.09) | 3.77 (2.94-4.82) |
| Trimesters 1 & 2 | 15/96 | 15.63 | 3.49 (1.69-7.21) | 2.44 (1.14-5.23) |
| Trimesters 1 & 3 | 13/65 | 20.00 | 4.72 (2.12-10.48) | 2.99 (1.25-7.16) |
| Trimesters 2 & 3 | 54/274 | 19.71 | 4.63 (3.13-6.85) | 3.50 (2.30-5.35) |
| Trimesters 1 & 2 & 3 | 134/753 | 17.80 | 4.08 (3.19-5.22) | 3.03 (2.32-3.95) |
| **Drug group** | | | | |
| Centrally acting | 26/136 | 19.12 | 4.36 (2.48-7.65) | 3.11 (1.70-5.72) |
| Beta blockers | 128/985 | 12.99 | 2.75 (2.16-3.52) | 2.36 (1.83-3.05) |
| Calcium channel blockers | 39/167 | 23.35 | 5.62 (3.51-9.00) | 4.73 (2.90-7.71) |
| >1 class | 209/896 | 23.33 | 5.61 (4.57-6.89) | 4.47 (3.59-5.56) |

**S4. Sub-analyses of low birth weight following in utero antihypertensive exposure. Table reports exposure to any antihypertensive started during the first trimester onwards, second trimester onwards and third trimester onwards, and exposure to centrally acting antihypertensive only, beta blocker only, calcium channel blocker only, or more than one class of medication. Adjusted for: Smoking status, SIMD quintile, diabetes, maternal BMI, parity, maternal age, alcohol use, previous stillbirths, drug misuse, preeclampsia ICD10 code and interactions (diabetes*gestation, age*SIMD, age*smoking, alcohol*drugs). RR = Risk ratio; 99% CI = 99% confidence interval; aRR = adjusted risk ratio.**

| **Low birth weight** | **Total**  **N=268,711** | **%** | **RR (99% CI)** | **aRR (99% CI)** |
| --- | --- | --- | --- | --- |
| No exposure | 11,051/250,693 | 4.41 | Reference | |
| **Trimesters** | | | | |
| Trimester 1 only | 56/439 | 12.76 | 3.17 (2.19-4.58) | 1.54 (0.89-2.66) |
| Trimester 2 only | 8/100 | 8.00 | 1.89 (0.73-4.87) | 0.51 (0.12-2.17) |
| Trimester 3 only | 159/768 | 20.70 | 5.66 (4.49-7.13) | 2.85 (2.00-4.05) |
| Trimesters 1 & 2 | 11/96 | 11.46 | 2.81 (1.23-6.41) | 1.05 (0.29-3.83) |
| Trimesters 1 & 3 | 12/65 | 18.46 | 4.91 (2.15-11.19) | 2.90 (0.81-10.45) |
| Trimesters 2 & 3 | 39/274 | 14.23 | 3.60 (2.30-5.62) | 1.52 (0.79-2.93) |
| Trimesters 1 & 2 & 3 | 124/753 | 16.47 | 4.27 (3.31-5.51) | 2.59 (1.77-3.78) |
| **Drug group** | | | | |
| Centrally acting | 19/136 | 13.97 | 3.43 (1.82-6.50) | 1.50 (0.56-4.00) |
| Beta blockers | 134/985 | 13.60 | 3.33 (2.62-4.24) | 2.46 (1.75-3.45) |
| Calcium channel blockers | 26/167 | 15.57 | 3.90 (2.25-6.76) | 1.24 (0.55-2.78) |
| >1 class | 188/896 | 20.98 | 5.62 (4.54-6.95) | 2.45 (1.77-3.38) |

**S5. Sub-analyses of small for gestational age following in utero antihypertensive exposure. Table reports exposure to any antihypertensive started during the first trimester onwards, second trimester onwards and third trimester onwards, and exposure to centrally acting antihypertensive only, beta blocker only, calcium channel blocker only, or more than one class of medication. Adjusted for: Smoking status, SIMD quintile, diabetes, maternal BMI, parity, maternal age, alcohol use, previous stillbirths, drug misuse, preeclampsia ICD10 code and interactions (diabetes*gestation, age*SIMD, age*smoking, alcohol*drugs). RR = Risk ratio; 99% CI = 99% confidence interval; aRR = adjusted risk ratio.**

| **Small for gestational age** | **Total** | **%** | **RR (99% CI)** | **aRR (99% CI)** |
| --- | --- | --- | --- | --- |
| No exposure | 24,412/252,598 | 9.66 | Reference | |
| **Trimesters** | | | | |
| Trimester 1 only | 57/439 | 12.98 | 1.46 (0.99-2.16) | 1.46 (0.97-2.18) |
| Trimester 2 only | 9/100 | 9.00 | 0.88 (0.32-2.40) | 0.94 (0.34-2.62) |
| Trimester 3 only | 125/768 | 16.27 | 2.03 (1.56-2.63) | 2.51 (1.93-3.31) |
| Trimesters 1 & 2 | 10/96 | 10.42 | 1.06 (0.41-2.74) | 1.16 (0.43-3.09) |
| Trimesters 1 & 3 | 6/65 | 9.23 | 0.97 (0.29-3.21) | 1.21 (0.35-4.13) |
| Trimesters 2 & 3 | 26/274 | 9.49 | 1.07 (0.61-1.87) | 1.45 (0.82-2.58) |
| Trimesters 1 & 2 & 3 | 125/753 | 16.60 | 1.99 (1.52-2.60) | 2.72 (2.06-3.58) |
| **Drug group** | | | | |
| Centrally acting | 10/136 | 7.35 | 0.74 (0.32-1.73) | 1.23 (0.52-2.89) |
| Beta blockers | 160/985 | 16.24 | 1.81 (1.45-2.27) | 2.22 (1.77-2.80) |
| Labetalol | 96/680 | 14.12 | 1.54 (1.16-2.04) | 1.92 (1.43-2.57) |
| Atenolol | 24/95 | 25.26 | 3.16 (1.72-5.81) | 3.83 (2.03-7.21) |
| Bisoprolol | 20/106 | 18.87 | 2.17 (1.15-4.12) | 2.67 (1.39-5.14) |
| Calcium channel blockers | 18/167 | 10.79 | 1.13 (0.59-2.15) | 1.24 (0.64-2.39) |
| >1 class | 142/896 | 15.85 | 1.76 (1.39-2.23) | 2.37 (1.86-3.03) |
